# The recovery of homicidal people diagnosed with schizophrenia and schizoaffective disorder—An interpretative phenomenological analysis

**DOI:** 10.3389/fpsyt.2022.951678

**Published:** 2023-01-19

**Authors:** Asztrik Kovács, Bence Ladányi, Noémi Farkas, Laura Stempel, Dániel Kiss, Évi Bittermann, József Rácz

**Affiliations:** ^1^Doctoral School of Psychology, Eötvös Loránd University, Budapest, Hungary; ^2^Institute of Psychology, Eötvös Loránd University, Budapest, Hungary; ^3^Department of Criminal Investigation, Hungarian National Police Headquarters, Budapest, Hungary; ^4^Szentgotthárd Psychiatric Home, Szentgotthárd, Hungary; ^5^Department of Addictology, Faculty of Health Sciences, Semmelweis University, Budapest, Hungary

**Keywords:** homicide, schizophrenia, recovery, forensic recovery, identity recovery, interpretative phenomenological analysis, long-term psychiatric care

## Abstract

**Objective:**

Identity recovery in people diagnosed with schizophrenia who have committed homicide poses several difficulties. Premorbid mental illnesses, the experience of psychosis, and the absence of cohesive ego functions may result in the inability to integrate the homicidal act into self-identity. Problems with integration increase the risk of recidivism and further mental problems. The aim of the present research was to explore how homicidal people diagnosed with schizophrenia make sense of their actions, and how they identify with the homicide.

**Method:**

Six semi-structured interviews were conducted at a long-term psychiatric home with people who had committed homicide and who had been diagnosed with schizophrenia. The interviews were transcribed verbatim and analysed using interpretative phenomenological analysis (IPA), an idiographic method rooted in phenomenologist traditions that focuses on how participants experience and make sense of events in their lives, and how those events affect their identity and sense of self.

**Results:**

Three personal experiential themes were established as a result of the analysis: (1) homicide and responsibility; (2) homicide and self; and (3) control over threats to self and self-evaluation. (1) Homicide was often reported to have been committed in a non-conscious, delusional state that may have led to the loss of self-determination. (2) Our interviewees struggled to integrate their acts into their identities. They distanced themselves from the crime or held multiple, parallel interpretations of the act. (3) Recovering patients experienced the constant threat of entering into a delusional reality and losing control. The importance of control was central to their self-evaluation. The patients appeared to distance themselves from the homicidal act and to regard their delusional selves as a threat to their lives.

**Conclusion:**

Therapy aimed at bolstering self-control, supporting the integration of the fragmented self, and raising awareness of the connections between delusional reality and standard, intersubjective reality may be helpful in reducing the instability of the self. Therapy aimed at processing complex grief and loss of family is also needed.

## 1. Introduction

### 1.1. Homicide and schizophrenia

Although different countries have different legal standards, it is widely accepted that diseased/altered mental functioning can affect the degree of individual criminal responsibility (1). Criminal responsibility and the legal concept of sanity are affected by the cultural and legal traditions of the given country. Countries that follow a common law model (the United States, Canada, the United Kingdom, Australia, and New Zealand), for example, apply a dichotomous approach based on the presence or absence of criminal responsibility. Other countries (such as the Netherlands, Belgium, and Germany) differentiate among various degrees of criminal responsibility (2). In Sweden, mental illness does not preclude someone from being found guilty, although their sentence may involve compulsory medication and treatment (2). In Hungary, Section 17 (3) of the Criminal Code states that “A person shall not be liable to punishment if he commits the punishable act in a state of mental disorder that renders him unable to recognise the consequences of his actions or to act according to such recognition.” This means that if a person is found to be unable to make sense of the crime and its consequences during the forensic psychiatric evaluation because of mental illness, they cannot be prosecuted. However, they can be found incapable, placed in the custody of a guardian, and sentenced to compulsory medication and psychotherapy.

Most individuals who cannot be found guilty due to mental illness are diagnosed with schizophrenia (4). The incidence of violent crime has been found to be higher among people with schizophrenia spectrum disorders than in the general population (5–7). The Diagnostic and Statistical Manual of Mental Disorders, Fifth Edition (DSM-5) states that although hostility and aggression are associated with schizophrenia, random assaults are rare (8). Nowadays, factors other than schizophrenia are claimed to predispose to crime, such as life events (physical abuse, parents’ criminal past, genetic factors), other dispositional factors (young age, male gender, lower income), or comorbid drug abuse (9–11). Researchers have found that crime is more common among people with mental illness who do not receive adequate treatment (12), whose pharmacotherapy is interrupted (13), or who actively experience delusions, paranoia (14), or hallucinations (6, 15, 16). The appropriate therapy and possible recovery of such patients pose many questions. Homicide is a traumatic event even in the life of a non-schizophrenic person, who will struggle to accept it as part of their life and accept responsibility for it (17). Understanding how patients with schizophrenia who have committed homicide make sense of their act years after the homicide provides valuable information for the planning of adequate treatment.

### 1.2. Recovery model

From a clinical perspective, recovery is a complete return to premorbid levels of functioning (18), or full functioning (8, 19). Successful recovery consists of vocational reestablishment and the regaining of social relationships, independent functioning, and self-integrity. The goal of the recovery model in psychology is to help patients regain their health and mental well-being after disease, trauma, crisis, or addiction by integrating the traumatic event and its physical and mental impacts into the patient’s self-identity (20–23). The recovery of identity is aimed at re-establishing a sense of the continuity, coherence, and sameness of the self (24).

### 1.3. Recovery after homicide

In the past decade, the recovery paradigm has attracted attention among forensic psychologists and psychiatrists (25–33). Adshead et al. referred to life after homicide as “life after death” for offenders [(34), p. 9]. Homicide leads to stigmatisation and self-stigmatisation; affects psychosocial circumstances, identity, and the concept of self in many ways; and has severe emotional and mental health consequences (35). The recovery process involves desisting from offending behaviour (28, 36), acknowledging the intra- and interpersonal circumstances of homicide (17), and accepting its consequences in personal and social life. Taking responsibility for the lifelong impact of homicide and facing feelings of loss and grief is of key importance in risk reduction (37). Making sense of the crime, understanding the reasons and motivations behind it, and creating a relevant narrative help to prevent more serious mental problems (18) and redefine the self by the integration of the act of homicide (17, 36). Indeed, addressing the coherence, continuity, and content of identity is central to forensic recovery (38).

During recovery, previous offenders report a change in their sense of self and a shift to a pro-social identity (39). The acceptance of responsibility is manifested in the emergence of an ex-offender identity and the acknowledgement of agency in the narratives of individuals who are in the process of forensic recovery (35, 40, 41). This change typically appears in the form of a shift from phrases such as “I didn’t do it” and “I did it, but I was mentally ill” to “I did it” (42). Patients tend to acknowledge that, despite their mental illnesses or drug abuse, they are responsible for the assault (35). Re-establishing the stability and wholeness of identity is one of the aims of forensic recovery, although little is known about the identity processes of people with a mental illness who have committed a crime (43).

### 1.4. Recovery of people diagnosed with schizophrenia

The definition of recovery as a complete return to full functioning (8, 16) does not apply to recovery from schizophrenia, as the illness appears in adolescence or early adulthood before social patterns and functioning are fully developed and independent (44). The criteria for recovery from schizophrenia have, thus, been revised to the sustained [i.e., longer than 2 years (45, 46)] remission of symptoms, social functioning (47), productivity, independent functioning, and optimism about the outcome from schizophrenia (46, 48, 49). Many clinical studies have found that, according to these criteria, recovery from schizophrenia does occur (50–55). However, the recovery of self is a difficult question in the context of schizophrenia.

### 1.5. The sense of self in schizophrenia

In a psychotic state, it is extremely difficult to maintain a sense of the congruence and continuity of the self and reality (56), which hinders the individual’s ability to acknowledge the intrapersonal and interpersonal circumstances and consequences of the crime they have committed. Psychotic delusions engender an altered sense of reality (57). Feyaerts et al. (57) distinguished two realities in schizophrenia: *delusional reality* and *standard reality*. Standard reality is the world of social norms and physical rules, while delusional reality is “a private framework that violates spatio-temporal and non-contradiction constraints of the intersubjective world” [(58), p. 1514]. Delusional reality may be experienced differently from standard reality in terms of sensual aspects, meanings, and contingency (57, 59, 60). Delusions may appear inadequate and inappropriate from an outsider’s perspective, but to the insider, they are experienced as adequate aspects of reality (57, 61). Karl Jaspers (59) described the delusional atmosphere as a different sense of reality in which everything has a new meaning. When this reality is questioned by society, the psychotic person tends to react with suspicion and self-protection (62).

Coping with the experience of different realities demands attempts at integration and defence. People with schizophrenic symptoms have immature defence mechanisms, such as negation, repression, fantasy, and splitting (63) or delusions (64). The concepts of splitting and disruption in the sense of self in schizophrenia are as old as the concept of schizophrenia itself (65).

Primitive defence mechanisms may lead to a disembodied, “unreal” sense of self, which is differentiated and alien from the rest of the world (66). Another possible consequence is so-called double bookkeeping (58), meaning that a person has experiences from both realities, but instead of creating consistency between them, the person experiences these realities as two isolated worlds with their own logic, sense, rules, and meanings. It also means that there are two senses of self in the two different realities.

Division of the self appears in the narratives of people diagnosed with schizophrenia. Life narratives of patients with schizophrenia have been found to be less coherent and elaborate than the narratives of a sine morbo control group (67). Patients with schizophrenia have also been found to have difficulties in extracting meaning from past events, which indicates the disturbance of the autobiographical self (68).

The experience of psychosis has a strong impact on the sense of self. Following a psychotic episode, patients have reported undergoing a process of self-criticism, distress, and anxiety in relation to their psychosis (69). Accepting the psychotic episode, integrating it into the sense of self, and regarding it as an opportunity for personal growth resulted in empowerment and contributed to recovery (69). Therapy aimed at restoring a sense of self-unity in schizophrenic people helps them to establish a positive and integrated sense of self, contributes to their social relationships, and helps them to build hope and invest in the future (70).

### 1.6. Homicide and schizophrenia

Given that the recovery model emphasises the importance of re-establishing a coherent sense of identity, while even non-criminals with schizophrenia have difficulties creating a coherent sense of identity (58, 66) and recovery is challenging even for sine morbo offenders (42), the question arises as to what happens to the sense of self in patients who are diagnosed with schizophrenia after committing homicide.

There is little information in the literature about how people who are diagnosed with schizophrenic spectrum disorder make sense of homicide. The goal of our study was to investigate how homicide affected such patients’ sense of self, and the progress they were able to achieve in terms of making sense of the traumatic event in their lives.

## 2. Materials and methods

We conducted six semi-structured interviews at a large psychiatric home. Ethical permission was given by Markusovszky Hospital Regional and Institutional Committee of Science and Research Ethics and the study was approved by the director of the institution. Written informed consent was obtained from the participants’ legal guardians for the publication of any potentially identifiable images or data included in this article. The procedure used to select the interviewees began by contacting the director of the institution with our inclusion criteria. The director then selected individuals who fitted our criteria and asked them if they were interested in taking part in the research. If they expressed willingness, she then obtained permission from their guardians for them to participate.

### 2.1. Sample

Our interviewees were patients who had been diagnosed with schizophrenia or schizoaffective disorder and who had committed homicide and been found to be not fully responsible for their crimes because of their mental illness (Table 1). We decided to include one person diagnosed with schizoaffective disorder, as psychosis and delusions are symptoms that are common to this disorder and schizophrenia (8). The participants had been sent to a forensic psychiatric institution after committing homicide and had become residents of the institution after being sentenced.

**TABLE 1:** Participants.

Pseudonym	Dávid	Márk	János	Ádám	Dániel	Ferenc
Sex	Male	Male	Male	Male	Male	Male
Age range	40–55	40–55	40–55	40–55	30–40	40–55
Education level	Higher education	Secondary education	Secondary education	Primary education	Primary education	Primary education
Age when homicide was committed	20–30	20–30	20–30	20–30	>20	30–40
Years spent in a forensic psychiatric institution	10–20	5–10	5–10	10–20	5–10	>5
Years spent in a psychiatric home	>5	5–10	10–20	10–20	5–10	10–20
Diagnostic category	Schizophrenic spectrum	Schizophrenic spectrum	Schizophrenic spectrum	Schizophrenic spectrum	Schizophrenic spectrum	Schizophrenic spectrum
Victim	Family member	Acquaintance	Family member	Family member	Family member	Family member

In four cases, a detailed diagnosis with multifaceted risk assessment had been made at the forensic psychiatric institution after the homicide, and during the treatment of patients, while two of the participants, Dávid and Márk, had received their diagnosis prior to the offence. As Dávid and Márk had been declared to have committed homicide during a psychotic episode, the timing of their diagnosis before or after the homicide was not of key importance to our research question. Our interviewees had been diagnosed again prior to their transfer from the forensic psychiatric institution to the psychiatric home for permanent care. Having entered the psychiatric home for permanent care, their physical and mental health is reviewed annually, including a diagnostic review in case new symptoms arise, although the main psychiatric diagnosis (schizophrenia and schizoaffective disorder) is usually upheld. Our participants had been diagnosed with paranoid schizophrenia, residual schizophrenia, unspecified schizoaffective disorder, paranoid schizophrenia, and hebephrenic schizophrenia. No comorbid mental illnesses were mentioned in their healthcare reports.

The offences had taken place between nine and 30 years prior to the interview. At the time of the interview, the participants were obliged to take psychotropic and neuroleptic medication and had not had a psychotic episode for a minimum of 2 years prior to the interview.

### 2.2. Procedure

We conducted one interview with each of our interviewees. The interviews were conducted by the third and fourth authors. Before the data collection, the interviewers were given training by their supervisors (the first and last authors, who are experienced researchers in the field of interpretative phenomenological analysis) on interview methodology, interview style, interview ethics, and establishing rapport. The structure of the interviews was designed jointly by these four authors. Each interview started with descriptive and narrative questions about the participant’s life within the institution, their diagnosis, and the homicidal offence. The interviewers asked questions about the impact of the crime on the participant’s life, their identities, and their personal meanings.

The interviews lasted between 20 min and 1 h and were carried out in a large psychiatric home between 2018 and 2020. In accordance with the ethical guidelines, the interviews were voluntary and participants were free to stop the interview, refuse to answer questions, or withdraw their participation during the interview. One of the participants stopped the interview after 20 min but did not withdraw his participation. Despite its brevity, this interview yielded important information about homicide and the concept of self, which convinced us to keep it in the research.

The interviews were recorded and transcribed verbatim, and interpretative phenomenological analysis (IPA) was carried out.

### 2.3. The phenomenological perspective

The exploration of such a deeply subjective and intrapsychological question requires an idiographic and inductive approach. Qualitative approaches, especially IPA, are a suitable method (71) because of their sensitivity and focus on the personal perspective. Based on its phenomenological and hermeneutical roots, IPA considers the self and experience as an inseparable unity (72). There is no self without experience and no experience without self. It is the intentional relationship between the self and its experience that can be examined (73). Psychological experiences can be understood only through the personal relevance and perspective of those who undergo them. Because of its epistemological grounding, the IPA method is congruent with our research and provided rich and in-depth knowledge grounded in the lived experience of homicidal patients diagnosed with schizophrenia.

### 2.4. Data analysis

We chose to apply IPA, as this method focuses on how participants experience and make sense of events in their lives, and how this affects their sense of self (71–73). In IPA, a double hermeneutical approach is used, in which the researcher interprets the participants’ meaning-making processes.

Maintaining a focus on the personal perspective, IPA is a two-step analytical process comprising idiographic analysis and cross-case analysis (72). During the idiographic analysis, the first four authors independently analysed the interviews one by one. This involved coding the interviews and creating interpretations of the interviews that were relevant to our research question.

A three-column method was used: the middle column contained the interview text, and the right-hand column contained the codes that constituted the pre-analysis. These codes were based on the psychological, linguistic, and social information in the interview text. The left-hand column contained experiential themes in the form of interpretations by the interviewers of experiences, meaning-making, and sense of self. Between 50 and 90 interpretations were generated from each interview.

The first four authors then independently categorised the experiential themes in the left-hand column interview by interview to identify those themes that covered the personal relevance of the experience.

Three to five personal experiential themes were identified in each interview and a table was created containing the interpretations and the original quotations on which the interpretations were based. The first four authors then discussed the interpretations until they reached a common understanding of the interviews and a consensus on the idiographic analysis. We established interrater reliability by means of independent coding, analysis, and consensus.

In the cross-case analysis, we compared the six idiographic analyses of the interviews, identified the common themes, merged those that were relevant, or created new themes based on the new perspectives that emerged from the comparison. According to the consensus reached by the authors, three personal experiential themes were identified, which are presented in the results section. The themes are personal, as they represent subjective, idiographic perspectives; and they are experiential since they are rooted in lived experience (72). The three themes answer our research question: *How do homicidal people diagnosed with schizophrenia make sense of their offence years after the homicide? How does it affect their sense of self?*

The coding was carried out in Google Sheets, as this provided the option to supervise each other’s study and to work simultaneously on the same document and add comments.

We then wrote up our results based on these themes and subthemes, relying on the interview texts, the information contained in the codes in the lefthand column, and the interpretations in the righthand column.

## 3. Results

The research group established five subthemes arranged according to the following three personal experiential themes: (1) homicide and responsibility; (2) homicide and self; and (3) control over threats to self and self-evaluation (Table 2).

**TABLE 2:** Personal experiential themes and subthemes.

Personal experiential themes	Subthemes
Homicide and responsibility	Avoiding abuse
	Influence of others
	Effect of psychosis
Homicide and self	Homicide is alien to the self
	Stupid mistake
	Tragedy
Control as basis of self-evaluation	

### 3.1. Homicide and responsibility

In all the interviews, the crime was depicted as having been committed in an altered state of consciousness, with a sense of loss of control, and under the influence of mental illness or abuse. The homicide was imputed to external factors and powers, implying that the patients were not merely offenders but also victims of the homicide.

#### 3.1.1. Avoiding abuse

*The only thing that was going through my head was not getting beaten that night. And that’s why I did it then.* (Dániel)

Dániel explained the homicide as being the result of continuous abuse at home. Due to his extreme sense of intimidation and vulnerability, his thoughts had been narrowed down only to avoid a beating. When avoidance became impossible, it led to an emotional outburst in the form of violence. His account includes agency and responsibility, as he says: “I did it then.” However, his emphasis on the circumstances indicates that the homicide was not experienced as a free, personal choice.

#### 3.1.2. Influence of others

*The pagans did bad to me and I went nuts and I made trouble.* (Ferenc)

Ferenc stated that his insanity was incited by others. He presents himself as the first victim of the “pagans,” who directed him to take the life of his grandmother.

Another interviewee (Ádám) offered a similar explanation, claiming that “satanists” had put “evil speech” into his mind that had driven him to commit homicide.

#### 3.1.3. The effect of psychosis

Our interviewees tended to talk about the circumstances of the crime. Schizophrenic symptoms, such as hallucinations and delusions, were considered to be of key importance in precipitating the crime. Dávid considered the crime to be the “fulfilment of schizophrenia.”

*I was already ill and I had these stupid thoughts.* (Márk)

For Márk, being ill meant having different, or *stupid*, thoughts. He depicted himself as a container for thoughts and the illness as a collection of irrational thoughts. His act was a consequence of the thoughts generated by the illness. In this way, he separated himself from the thoughts that had guided him to commit the homicide. Interestingly, a sense of being “mentally ill” did not appear in his account. He experienced the illness as taking control over his body. János reported an experience of a similar kind:

*Suddenly, from one day to the other, I started to hear voices saying “Kill her, stab that woman” and “she is not your mother”.* (János)

János indicated that he had had no preparation for dealing with the voices, which seemed extremely convincing. They not only commanded him to commit homicide, even specifying the method to be used but also weakened his ability to resist by claiming that the *woman* was not his *mother*. The influence of the illness was still regarded as the main cause of the homicide, even decades later.

*I know that it was my fault. It was my fault because I didn’t take the medicine, which is the biggest mistake in this illness. (Dávid)*

Dávid pointed out that by failing to take his medication he was responsible for letting schizophrenia take control of him and for committing the homicide. He seemed to suggest a balance between maintaining a normal self-image that is not homicidal while still being responsible for the homicide—or, more precisely, for controlling the “murderer illness.”

The interviewees inferred that the homicide had been committed while experiencing a distorted sense of reality. At the time of the interview, this reality was explained as a false or non-egosyntonic reality. The participants’ thoughts, motivations, and feelings were no longer understandable to them. Importantly, at the time of the interviews, the participants had not experienced psychosis for at least 2 years. They explained psychosis as something that took total control over their actions and thoughts. Although the manner of this influence varied across the interviews, the sense of being a victim of the illness was a common experience.

### 3.2. Homicide and self

Our interviewees described their crime as an unintentional act, and even several years after the homicide they either denied their connection with the crime or struggled to interpret and integrate it. The multiple interpretations of the homicide created constant tension within the concept of the self, making it a struggle to create a stable sense of self. This was manifested in alienation and a sense of guilt, grief, and loss.

#### 3.2.1. Homicide as alien to the self

Most of our interviewees tended to consider their crime as incompatible with themself. Ádám, for example, appeared to be unaware of his crime “What did I do? I still cannot imagine.” Even those who were able to speak about their crime found it difficult.

*Well, I would rather not mention it, because you wouldn’t believe it. You wouldn’t believe that I did such a thing, which happened.* (Ferenc)

Ferenc refused to speak about the homicide because he considered it incompatible with himself. By repeating the phrase “you wouldn’t believe” he involved the interviewer in his struggle to integrate the homicide. He thought that the interviewer, who was meeting him for the first time, would find it impossible to believe he had committed such a crime. This specific and dynamic relationship with his crime is manifested in the transition from the verb “I did” to “happened” by the end of the sentence. The experience of the fragmented self is derived not from an egosyntonic act but from an event to which the person was subject.

#### 3.2.2. Stupid mistake

*I made a huge stupid mistake which*… *I still can’t understand in my normal senses what this whole thing was. I would rather not talk about it at all.* (Dávid)

Dávid appeared to struggle to integrate the homicide into himself. This explains why he presented two interpretations of the crime: first, he considered it to be a “stupid mistake” that was his fault. He emphasised how, at the time of the interview, he was able to reflect on his act: he could not yet understand “what this whole thing was.”

#### 3.2.3. Tragedy

Some of our interviewees regarded themselves as victims of events. They described the crime and its consequences as a “rupture” or “tear” in their lives. Ferenc stated that “It was an accident, a tragedy for me,” which inevitably led to the loss of family relationships, freedom, and a healthy self-image.

*I didn’t even understand what I’d done, only when I got to the [forensic psychiatric] institution and they gave me the first pills. I came round and “Oh my God, you have killed your mother”. That came to my mind at first. And I condemned myself, but I still pray for her every night.* (János)

In this quotation, János indicated his shock at realising what he had done. After taking the medicine, his point of view changed. Previously, he had trusted the coercive voices in his head, perceiving his mother as a stranger who had to be killed, but this belief changed radically once he was in the hospital. Even the subject of his sentence changed: the first-person description becomes a second-person exclamation: “Oh my God, you have killed your mother.” This appears as a complete turnaround in an inner conversation, a switch from seeing himself as someone who committed a righteous act justified by the voices to finding himself the murderer of his own mother.

*Unfortunately, I did that, but I shouldn’t have done that. By the way, it was a homicide. Unfortunately, I deeply regret it and I will regret it till the end of my life.* (Dániel)

The conflict between regretting the homicide and acknowledging responsibility for the homicide was palpable even 18 years after the event. Dániel placed particular emphasis on expressing his regret and sorrow, attempting to distance himself from the homicide by using a formal phrase with no indication of agency: “by the way, it was a homicide.”

Even when patients with schizophrenia who have committed homicide struggle to make sense of the homicide, it has a strong impact on their self-concept. Homicide committed in a state of psychosis appears to be an act that cannot be fully integrated into the individual’s sense of self. It seems to create a fissure in the unity of the self. Although this fissure in the sense of self is not a new phenomenon in schizophrenia, homicide committed in a state of psychosis appears to amplify the threat represented by the delusional self. Homicide and its consequences force an awareness that cannot be made sense of in the context of standard, intersubjective reality. It creates an alienated, incomprehensible element in the individual’s sense of self. While it contributes to self-defence against the weight of grief and responsibility, the patient in recovery is in a continuous dynamic relationship with this part of the self.

### 3.3. Control: A basis for self-evaluation

Some of our interviewees described their state of mind when the homicide took place as being under the influence of another force, a state in which their act was righteous and logical. Their interpretation of the homicide subsequently changed, and it was considered to be the result of a loss of self-control in the context of the psychotic reality. Losing control and committing homicide had a powerful, multifaceted impact on our interviewees, regardless of the level of responsibility they acknowledged in their interviews. This impact on their sense of self and their relationship to the self was a central motif. Control had become a central aspect of self-evaluation for all interviewees.

*I know how to control myself, what is good and what is not good. And like this I don’t commit any crime.* (János)

Being aware of himself and his illness and maintaining control over them were essential elements in his self-evaluation, which provided him with a sense of agency. Being a criminal and committing a crime appeared as a constant threat while taking firm control of the self was of key importance.

*I don’t think I would go nuts because of the same things as back then. And anyway, to do such things I would need to get in a very very deep depression, so*… *I don’t want to be in depression, and I don’t think I will be (*…*). If somehow I will be and I will hurt someone, then I will rather wait one or two hours.* (Dániel)

Dániel claimed that his depression – or, more precisely, his inability to acknowledge and handle his “depression” in its early stages—played an important role in terms of committing his crime. He explained how he had gained awareness of his illness, which prevented him from losing control.

Self-control, in the form of taking medication, was mentioned in Dávid’s interview:

*The medication is the most important thing, that it is well regulated. (*…*) I am active in many senses, mental and physical, and I don’t let myself go. That is the worst, when one lets oneself go.* (Dávid)

Dávid emphasised the role of medication in staying well-balanced and in control. He praised himself for his activity and his self-care. Experiencing his ability to be in control of these aspects of his life was essential.

Maintaining control over their habits and emotions is fundamental to their sense of self in offenders with schizophrenia and schizoaffective disorders. As recovery takes place in standard reality, the act of homicide cannot be wholly understood. Patients are forced to alienate themselves from the homicide and its consequences. Alienation is difficult, however, given the embodied experience of the psychosis, with its altered thoughts and feelings. The threat of the illness is considered to be intrapersonal: a part of them is constantly threatening outbursts and violence. Constant control over their thoughts, emotions, and mental state, and seeing themselves as having a part that is dangerous and permanently threatening violence, is important if the patients are to gain a sense of competence, confidence, and self-trust.

## 4. Discussion

### 4.1. Summary of the findings

In our research, we explored how patients diagnosed with schizophrenic spectrum disorder experience and make sense of homicide. Our findings can be grouped according to three themes. First and foremost, the homicide was not a personal decision: the patients reported being commanded by an irresistible power. Second, all our interviewees struggled to integrate homicide into their identity. They were found to distance themselves from the crime or to hold parallel interpretations of the homicide. Third, self-control was central to their self-evaluation.

### 4.2. Identity recovery among homicidal people diagnosed with schizophrenia

The fissure between delusional and standard reality—that is, between the delusional and non-delusional self—seems to be a fundamental conflict for patients with schizophrenia who have committed homicide. For healthy people recovering from committing homicide, integrating the crime and acknowledging it as an “I did” act is of key importance in maintaining the continuity of the self, regaining autonomy, accepting the consequences of the homicide (17, 35, 37), and reducing the possibility of recidivism and more mental problems (74). In our study, the “I did” narrative and agency did not occur in some cases, as participants were aiming to distance themselves from the delusional self that had committed the homicide. Link and Stueve (75) found that patients experiencing psychotic episodes explained their violence as a reaction to “outside powers” and influences, which in our study was only part of the truth. Homicide committed in the context of delusional reality appeared to be difficult to make sense of later on, despite its significant impact on the perpetrators’ lives in standard, intersubjective reality.

Psychotic experiences are extremely difficult to make sense of, as they occur in the context of an altered state of mind, or delusional reality (57), and are experienced as alien or disjoint from the intersubjective sense of self (56). Decades after the homicide, those who have been able to find an explanation for and meaning in their psychotic episodes seem to recover more successfully (76).

We found that our participants struggled to integrate the fact of homicide into their personal selves. Distancing themselves from the crime, or having several co-existing interpretations of the homicide, appear to be strategies for finding meaning and defending the self from identification as an offender. Maintaining control over the illness has been shown to be important to patients, as it allows them to regain some of their autonomy and function at a higher level (77, 78).

For our participants, regarding themselves as mentally ill and considering their illness as at least partly to blame for the homicide appeared as a possible means of self-defence. However, even decades after the homicide, the topic of the offence was narrated with self-alienation or extreme regret. We found that an identity as mentally ill was established prior to the establishment of a homicidal identity. This may make it easier to maintain a sense of the self as innocent and to make sense of both the crime and the experience of schizophrenia, although, at the same time, it perpetuates difficulties in interpreting the homicide and accepting its consequences, such as loss of social relationships, long-term care and stigmatisation (17, 37), which put the patient at risk of depression, PTSD, and recidivism (74).

According to our findings, the delusional self appears to be experienced as a threat to the recovering self, and as a violent and aggressive being who is able to commit suicide. This violent self is, however, a part of the patient and has victimised them as well. Constant vigilance over mental illness and distrust of the self was observed in the interviews, which elevated the importance and centrality of self-control in self-evaluation. It suggested that some responsibility had been taken, even if it was verbalised differently. Constant control infers a constant sense of threat and hostility within the participants’ self-concept. Constant vigilance and control, however, threaten psychological exhaustion, loss of control, and a fixation on the sense of intrapersonal distrust.

### 4.3. Limitations

The interviews were carried out by researchers from the (blinded) university with the help of the director of the psychiatric home, (blinded). The director helped to select the interviewees and guided them to the interview. The director’s knowledge of the institutional context and the residents had both advantages (a profound knowledge of the lives and backgrounds of the residents, contextual knowledge of the staff and the background of the psychiatric home) and disadvantages (the distorting influence of pre-existing knowledge). Power differences between the interviewees and interviewers may have affected the interviewees’ accounts, as the service users may have wanted to meet the interviewer’s requirements. The socioeconomic gap between the interviewees and interviewers may also have hindered deep understanding. Some of the participants’ narratives appeared fragmentary, which may have been due to cognitive impairments, the effects of long-term care, schizophrenia itself, and the side effects of medication. There were also barriers in terms of understanding and interpreting the narratives. Given the sensitivity of the topic, the interviewers had to be careful to respect the patients’ boundaries and allow them to share only as much as they intended. Because of the strict rules in the institution and difficulties obtaining access, only one interview could be conducted with each interviewee. Given the small size of the available sample of homicidal people diagnosed with schizophrenia, only six interviews could be conducted. However, the number of patients and the depth of the interviews proved to be sufficient for exploratory research.

In the present study, we focussed on how the participants made sense of the act of homicide and the nature of the intrapsychological processes. For this reason, and to maintain clarity, we were obliged to restrict our findings section and have not presented those parts that referred to the consequences of family relationships. A discussion of these consequences would require a separate article, as social support is essential for a successful recovery and the successful integration of various realities and selves. In most cases, the victim of psychotic homicide is a family member, which has severe impacts on family relationships. The consequences of committing homicide, which include loss of social contacts, complicated grief after murdering a relative, and stigmatisation, result in extreme stress and threat to the self (57). Healthcare workers also tend to fear and stigmatise patients who have committed homicide, resulting in avoidance and a lack of therapeutic opportunities (56).

Our research involved interviews with six patients, from which we were able to obtain a complex picture. However, a longitudinal interview study might contribute to an understanding of the process that the patients go through following the homicide. Our ethical permission and difficulties in accessing this population, followed by the restrictions imposed during the COVID-19 pandemic, imposed limitations on our research. We believe that the detailed information and first-person accounts provide a basis for further research on criminals with schizophrenia and offer help to practitioners in understanding and focussing on meaning-making, sense of control, and sense of self among offenders diagnosed with schizophrenia.

### 4.4. Conclusion and implications

Homicidal people with schizophrenia struggle to accept the unacceptable and integrate their acts into their concept of the self. They experience homicide as a tragedy in their lives and as a rupture in their psyche. They seem to experience extreme guilt and anxiety when confronting their homicide. Our interviewees, who were homicidal patients diagnosed with schizophrenia and schizoaffective disorder, seemed to struggle to make sense of being mentally ill and homicidal. Our results demonstrated how the concept of responsibility emerged in connection with maintaining control over psychosis. Our interviewees presented many interpretations of the same experience, which apparently confirms the instability of the self and self-mistrust. This may be an indication of improved mental health since the individual now accepts that they may not be experiencing intersubjective reality and cannot trust their delusional selves. Mental illness is a constant threat over which they have to maintain control, and this becomes the core of their self-evaluation. Acknowledging the difficulties in recovery among offenders with schizophrenia, reconstructing the concept of the self and the meaning of and relationship with mental illness and homicide constitute the main therapeutic focus and the focus of future research. However, this would still not ensure full recovery: the patients would remain under legal guardianship and in a psychiatric home, although the psychological impacts of homicide and mental illness would be alleviated.

As the victim of psychotic homicide tends to be a family member, helping patients to process their complicated grief and raising their awareness of the homicide and mental illness may help them regain a sense of agency and autonomy and prevent recidivism. The development of self-control could be supported by identifying appropriate psychological and social resources and by providing vocational opportunities.

According to our participants, they did not receive therapy focusing on processing the homicide and losing their families either in the forensic psychiatric institution or in the permanent care home. Instead, they were advised to “look forward” and “start a new life.”

Helping healthcare workers to overcome their fear of patients who have committed homicide might provide opportunities for the patients to process the trauma of the homicide and acknowledge its consequences (79). Exploring the experiences of professionals who treat homicidal people may provide important information that will contribute to overcoming fears and therapeutic difficulties.

## Data availability statement

The raw data supporting the conclusions of this article will be made available by the authors, without undue reservation.

## Ethics statement

The studies involving human participants were reviewed and approved by Markusovszky Hospital Regional and Institutional Committee of Science and Research Ethics, Szombathely, Hungary. Written informed consent was not provided by the participants, as they were not legally capable, but it was provided by their legal guardians.

## Author contributions

AK contributed with the planning of interviewing process, research methodology, acted as a coder, identified the relevant literature, and wrote the manuscript. BL made significant contribution in analysing the interviews. NF and LS collected the interviews at Szentgotthárd Psychiatric Home, and contributed in the coding process. ÉB helped in finding the interviewees in the institution and also contributed in the interpretation of the data. DK pointed out the incoherence in the first form of manuscripts and gave important comments on the text. JR supervised and planned the whole research project and commented and shaped the manuscript. All authors contributed to the article and approved the submitted version.
